# 1476. Optimizing Preoperative Antibiotic Administration Timing to Reduce Surgical Site Infections

**DOI:** 10.1093/ofid/ofad500.1312

**Published:** 2023-11-27

**Authors:** Heather Voss, Asra Salim, Mike Postelnick, Jaime Borkowski

**Affiliations:** Northwestern Memorial Healthcare, McHenry, Illinois; Northwestern Memorial Healthcare, McHenry, Illinois; Northwestern Medicine, Chicago, Illinois; NM Delnor Hospital, Geneva, Illinois

## Abstract

**Background:**

The administration of clinically indicated antibiotics prior to surgery in combination with other evidence-based practices reduces the risk of surgical site infection (SSI). A retrospective study of SSI at a community-based hospital that performs 5,700 procedures annually revealed broad variability in the timing of prophylactic antibiotic administration in relation to the time of incision. A Tableau dashboard was created to compare antibiotic administration to surgical incision timing and the impact variability may have on the incidence of SSI.

**Methods:**

A multidisciplinary team was formed with surgeons, anesthesia, nursing, antimicrobial and diagnostic stewardship, infection prevention, and analytics representatives. Antibiotics selected for inclusion were those most prescribed for surgical prophylaxis according to organizational practice guidelines. Using the dashboard, optimal administration times were identified based upon the pharmacokinetics of the antibiotic as well as the expected tissue concentration at the incision site when available. (See table) Surgical encounters that received an antibiotic were classified as “pass”, “fail”, or “other”. Antibiotics started within the determined timeframe passed, those started earlier or later than the timeframe failed, and the others received antibiotics not included in the dashboard logic.

**Figure:**
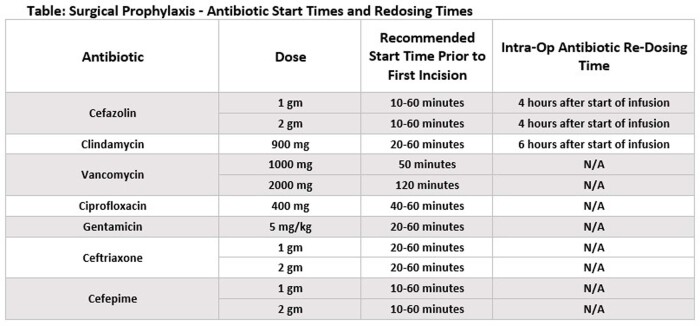

**Results:**

In a baseline review 35.5% of cases failed timing criteria. Failures occurred most frequently with cefazolin, ceftriaxone and clindamycin. A retrospective analysis of SSI (n = 34) classified using the National Healthcare Safety Network (NHSN) definitions correlated with the baseline data. The outliers were comprised of antibiotics started too close to or after incision (14.7%) and antibiotics started much earlier than the dosing timeframe and incision (17.6%).

**Conclusion:**

The surgical prophylaxis timing dashboard helped to identify gaps in administration practices and highlighted the need for process improvement initiatives. Ongoing interventions to improve adherence to national guidelines will be evaluated to determine the impact on SSI rates.

**Disclosures:**

**All Authors**: No reported disclosures

